# Disproportionation of Co^2+^ in the Topochemically Reduced Oxide LaSrCoRuO_5_

**DOI:** 10.1002/anie.202313067

**Published:** 2024-01-04

**Authors:** Zhilin Liang, Maria Batuk, Fabio Orlandi, Pascal Manuel, Joke Hadermann, Michael A. Hayward

**Affiliations:** ^1^ Department of Chemistry, Inorganic Chemistry Laboratory University of Oxford South Parks Road Oxford OX1 3QR UK; ^2^ EMAT University of Antwerp Groenenborgerlaan 171 2020 Antwerp Belgium; ^3^ ISIS Neutron and Muon Source Rutherford Appleton Laboratory Chilton Oxon OX11 0QX UK

**Keywords:** Disproportionation, Double Perovskite Oxides, Ferromagnetism, Topochemical Reduction, Transition-Metal Oxides

## Abstract

Complex transition‐metal oxides exhibit a wide variety of chemical and physical properties which are a strong function the local electronic states of the transition‐metal centres, as determined by a combination of metal oxidation state and local coordination environment. Topochemical reduction of the double perovskite oxide, LaSrCoRuO_6_, using Zr, yields LaSrCoRuO_5_. This reduced phase contains an ordered array of apex‐linked square‐based pyramidal Ru^3+^O_5_, square‐planar Co^1+^O_4_ and octahedral Co^3+^O_6_ units, consistent with the coordination‐geometry driven disproportionation of Co^2+^. Coordination‐geometry driven disproportionation of d^7^ transition‐metal cations (e.g. Rh^2+^, Pd^3+^, Pt^3+^) is common in complex oxides containing 4d and 5d metals. However, the weak ligand field experienced by a 3d transition‐metal such as cobalt leads to the expectation that d^7+^ Co^2+^ should be stable to disproportionation in oxide environments, so the presence of Co^1+^O_4_ and Co^3+^O_6_ units in LaSrCoRuO_5_ is surprising. Low‐temperature measurements indicate LaSrCoRuO_5_ adopts a ferromagnetically ordered state below 120 K due to couplings between S=^1^/_2_ Ru^3+^ and S=1 Co^1+^.

## Graphical abstract

Topochemical reduction of LaSrCoRuO_6_ yields LaSrCoRuO_5_ a phase containing Co^3+^O_6_, Co^1+^O_4_ and Ru^3+^O_5_ units, consistent with the coordination‐geometry‐driven disproportionation of Co^2+^ in an extended oxide.

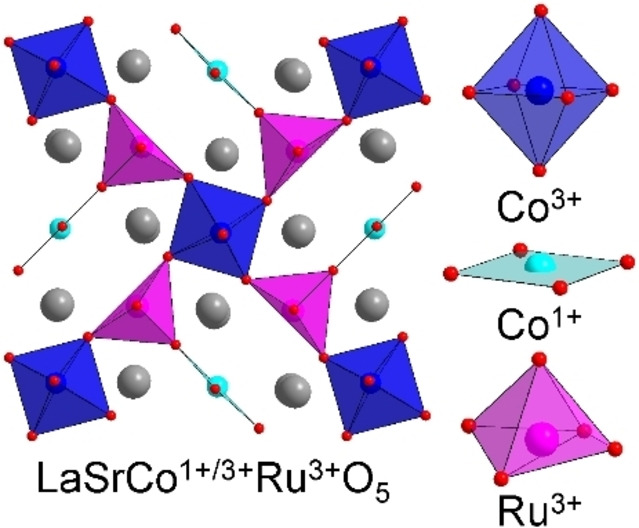

Complex metal oxides have been the subject of extensive study due to the wide variety properties they exhibit. These range from electronic and magnetic behaviors such as ferroelectricity, superconductivity and magnetoresistance to an extensive array of catalytic and electrochemical phenomena. As the chemical and physical behaviors exhibited by metal oxides tend to depend strongly on the electric configurations of the metal cations they contain (defined by a combination of oxidation states and coordination environments), there has been an enduring interest in establishing composition‐structure‐property relations in extended oxide systems to explore these features. These studies have revealed that a number of elements exhibit ‘disfavored’ oxidation states in oxide environments, i.e. oxidation states that appear to be thermodynamically accessible (sufficient lattice energy to overcome the required ionization energy) but are unstable with respect to disproportionation, when the metal is located in an extended oxide framework.

The instability of some of these disfavored states, such as the disproportionation of Pb^3+^ and Bi^4+^ in Pb_2_O_3_ and BiO_2_ respectively,[^1^, ^2^] can be accounted for by universal chemical concepts—in this instance the global instability of ns^1^ electron configurations in main group metals leads to Pb_2_O_3_ and BiO_2_ being better described as Pb^II^Pb^IV^O_3_ and Bi^III^Bi^V^O_4_ respectively.

However, similar disproportionations are observed in transition‐metal systems, where the instability of the metal oxidation state cannot be easily attributed to a global instability of a particular electron count but appears to arise from the favorability of particular combinations of d‐electron count and local coordination environment. For example, AgO is better described as Ag^I^Ag^III^O_2_,^[3]^ with the disproportionation of Ag^II^ attributed to the favorability of locating d^10^ Ag^I^ in a linear coordination and d^8^ Ag^III^ in square‐planar coordination sites within the oxide framework. Likewise, analogous disproportionations of d^7^ cations, such as Pd^III^ or Pt^III^ are observed, driven by the favorability of locating d^6^ Pd^IV^/Pt^IV^ cations in octahedral environments and d^8^ Pd^II^/Pt^II^ cations in square‐planar coordinations, in phases such as K_2_Pd^II^_3_Pd^IV^O_6_ and CdPt^II^Pt^IV^_2_O_6_.[^4^, ^5^]

Recently we observed the disproportionation of d^7^ Rh^II^ during the topochemical reduction of the Ruddlesden‐Popper LaSrM_0.5_Rh_0.5_O_4_ (M=Co, Ni) and perovskite LaM_0.5_Rh_0.5_O_3_ oxides, with the reduced phases (LaSrM_0.5_Rh_0.5_O_3.25_ and LaM_0.5_Rh_0.5_O_2.25_ respectively) hosting d^8^ Rh^I^ in square‐planar coordination sites, and d^6^ Rh^III^ in 5‐coordinate, square‐based pyramidal sites.[^6^, ^7^] Here we describe the first observation of the disproportionation of d^7^ Co^II^ in an extended oxide, which occurs during the topochemical reduction of the double perovskite oxide LaSrCoRuO_6_ to LaSrCoRuO_5_.

Previous work revealed that rapidly quenching the double perovskite oxide LaSrNiRuO_6_ through a *R*‐3 to *P*2_1_/*n* phase transition (*T*≈400 °C)^[8]^ increased the reactivity of this oxide phase with CaH_2_, allowing the preparation of the infinite layer phase, LaSrNiRuO_4_, by topochemical anion deintercalation.[^9^, ^10^] The corresponding cobalt phase, LaSrCoRuO_6_,[^11^, ^12^] exhibits an analogous phase transition at *T*≈450 °C. Rapidly quenching LaSrCoRuO_6_ through its *R*‐3 to *P*2_1_/*n* phase transition also enhances its reactivity enabling the preparation of the infinite layer phase LaSrCoRuO_4_ via reaction with binary metal hydrides, as will be described in detail elsewhere. However, in contrast to the LaSrNiRuO_6‐*x*_ system, quenched samples of LaSrCoRuO_6_ can be reduced to a phase of intermediate oxygen content (shown to be LaSrCoRuO_5_ by oxidative thermogravimetric analysis) by reaction with a Zr getter at 450 °C.

Synchrotron X‐ray powder diffraction (SXRD) data collected from LaSrCoRuO_5_ could be indexed using a body‐centered monoclinic unit cell (*a*=5.40 Å, *b*=5.41 Å, *c*=8.16 Å, γ=90.5 °) consistent with the retention of the perovskite framework from the LaSrCoRuO_6_ parent phase. However, close inspection revealed a series of weak additional reflections in the SXRD data that could not be indexed by this cell. Electron diffraction (ED) data collected from LaSrCoRuO_5_, shown in Figure 1, are consistent with a 2×2×1 cell expansion compared to the LaSrCoRuO_6_ parent phase (2√2×2√2×2 compared to a simple ABO_3_ perovskite unit cell).[^13^, ^14^] This expanded cell accounts for all the additional weak peaks observed in the SXRD data and can also index neutron powder diffraction (NPD) data collected at room temperature from LaSrCoRuO_5_.

**Figure 1:**
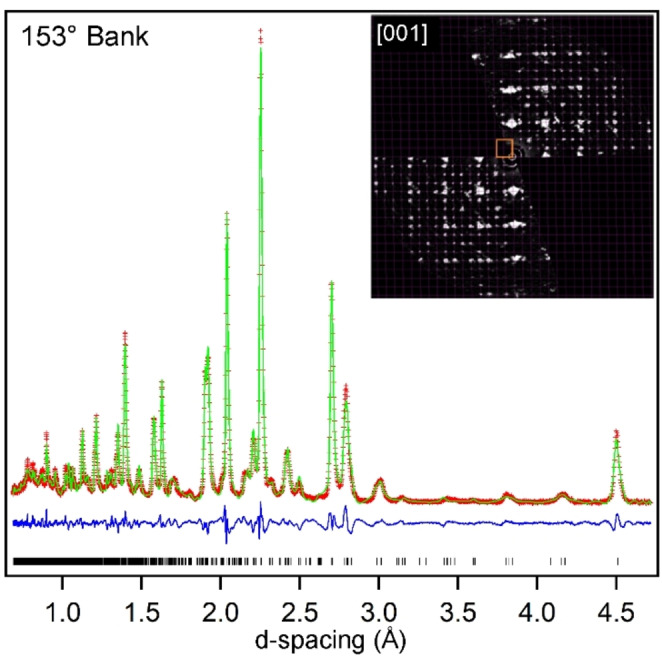
Observed, calculated and difference plots from structural refinement of LaSrCoRuO_5_ against NPD data. Inset shows ED pattern demonstrating 2√2×2√2×2 cell expansion.

Considering the A_2_BB'O_5_ composition and the 2√2×2√2×2 cell expansion of the phase, a number of anion‐vacancy ordered and B‐site cation ordered perovskite structural models were considered for LaSrCoRuO_5_. It was observed that a good fit to the SXRD and NPD data could be achieved using a model based on the anion‐vacancy ordered structure of LaNi_0.9_Co_0.1_O_2.5_ which consists of a network of apex‐linked 6‐coordinate octahedral, 5‐coordinate square‐based pyramidal and 4‐coordinate square planar BO_x_ units.^[15]^ The model was modified to take account of the rock salt ordering of the Co and Ru cations, so that the Ru centers were exclusively located within 5‐coordinate sites, while the Co centers occupied both 6‐ and 4‐coordinate sites within a monoclinic unit cell (*a*=10.8128(2) Å, *b*=10.8231(2) Å, *c*=8.1626(1) Å, γ=90.55(1) °) with *P*112_1_ space group symmetry, as shown in Figure 2. The model was refined against the NPD data to achieve a good fit (wRp=6.33 %) as shown in Figure 1 and described in detail in the Supporting Information.^[16]^

**Figure 2:**
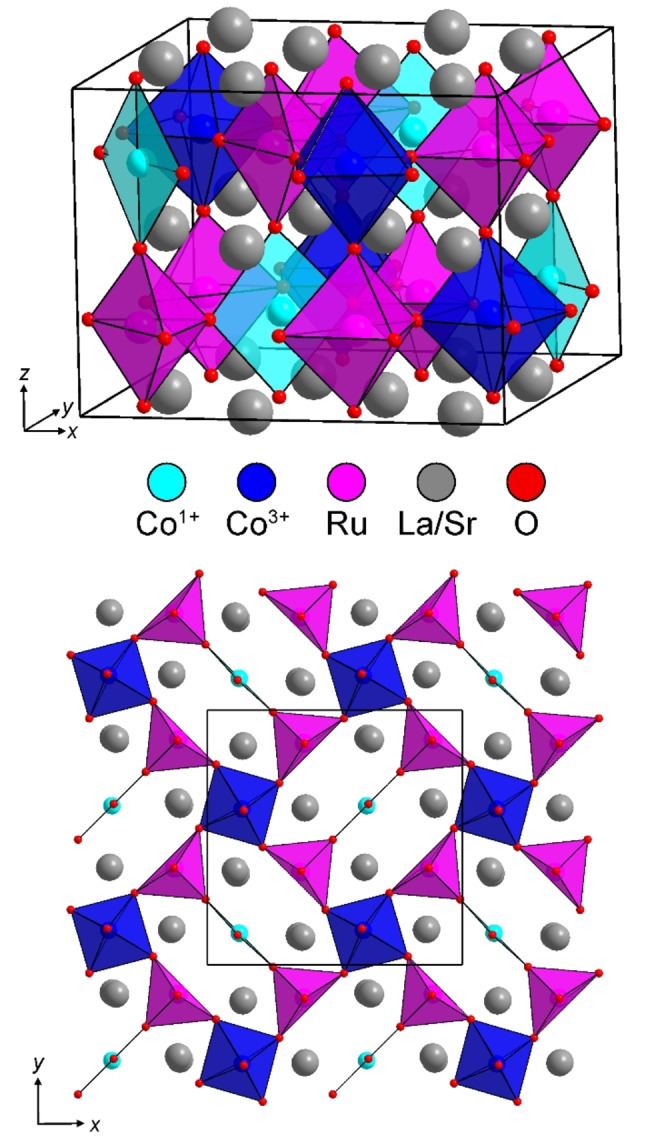
Crystal structure of LaSrCoRuO_5_ (top) and a projection of the transition metal coordination polyhedra at z≈0.75 (bottom).

The crystal structure of LaSrCoRuO_5_ shown in Figure 2 reveals that the cobalt cations occupy two distinct sites within the oxide framework. A 4‐coordinate planar site and a 6‐coordniate octahedral site. The location of the cobalt cations in two distinct sites is reminiscent of the local‐coordination‐driven disproportionation of transition metals with d^7^ electron counts observed for Pd^3+^ and Pt^3+^ and more recently Rh^2+^, and suggests the disproportionation of Co^2+^ into Co^1+^ (square‐planar) and Co^3+^ (octahedral). Analysis of the local coordination environments of the cobalt centers is hampered by the lack of reported Co^1+^O_4_ units for comparison. However, the observed bond lengths of the CoO_4_ units in LaSrCoRuO_5_ (Co−O=2.032(11) Å×2; 2.119(9) Å×2) are significantly longer than those in the Co^2+^O_4_ units reported in Sr_3_Co_2_O_4_Cl_2_ (Co−O=2.007(1) Å×4)^[17]^ or Sr_2_CoO_2_Cu_2_S_2_ (Co−O=1.995(1) Å×4)^[18]^ consistent with assignment of Co^1+^O_4_ for the units present in LaSrCoRuO_5_. Bond valance sums (BVS)^[19]^ calculated using parameters for Co^2+^ yield values of LaSrCoRuO_5_ : Co+1.42, Sr_3_Co_2_O_4_Cl_2_ : Co+1.70 and Sr_2_CoO_2_Cu_2_S_2_ : Co+1.76. The CoO_6_ units in LaSrCoRuO_5_ have a rather irregular shape but exhibit an average bond length of <Co−O=2.004 Å> (BVS=Co+2.69) compared to <Co−O=2.033 Å> (BVS=Co+2.38) the Co^II^O_6_ units in the LaSrCoRuO_6_ parent phase.^[12]^ Thus, it can be seen that the BVS values of the square‐planar (BVS=Co+1.42) and octahedral sites (BVS=Co+2.69) in LaSrCoRuO_5_ differ by 1.27 units. This difference is significantly larger than the difference between the octahedral and tetrahedral sites in the Co^2+^ brownmillerite phase La_2_Co_2_O_5_ (CoO_6_ BVS=Co+2.23; CoO_4_ BVS=Co+2.07, Δ=0.16)^[20]^ or the difference between octahedral and square‐planar sites in the Ni^2+^ phase La_2_Ni_2_O_5_ (NiO_6_ BVS=2.08; NiO_4_ BVS=2.11, Δ=0.03)^[21]^ and provides strong support for the disproportionation of Co^2+^ in LaSrCoRuO_5_.

In an attempt to further confirm the disproportionation of Co^2+^, cobalt EELS data collected from LaSrCoRuO_5_. These data show a single set of Co L_2_ and L_3_ peaks (Figure S14 in the Supporting Information) and thus represent the superposition of signals from both the square‐planar and octahedral cobalt sites. In the absence of a Co^1+^ oxide standard we are unable to know if a Co^1+^/Co^3+^ oxidation state combination would be expected to lead to a resolvable splitting of the L_2_ and L_3_ peaks. It should be noted that splitting of Co^2+^/Co^3+^ signals is not resolvable for Co_3_O_4_.^[22]^ The L_3_/L_2_ intensity ratio (4.83) and L_3_‐L_2_ energy difference (15.06 eV) from the data are broadly consistent with Co^2+^.

Magnetization data collected from LaSrCoRuO_5_ indicate that, in common with many other topochemically reduced phases containing cobalt, samples of LaSrCoRuO_5_ contain small quantities of ferromagnetic, elemental cobalt not detectable by diffraction. The magnetization of LaSrCoRuO_5_ was therefore measured using the ‘ferromagnetic subtraction’ method described in the Supporting Information. A plot of the magnetic susceptibility of LaSrCoRuO_5_ against temperature (Figure 3a) can be fit by the Curie–Weiss law in the range 140<T/K<300. However, the extracted Curie constant (C=3.76 cm^3^ K mol^−1^; θ=+82.7 K) is much larger than can be accounted for by a combination of Co^1+^, Co^3+^ and Ru^3+^ cations (even with the cobalt centers in high‐spin states), suggesting strong magnetic interactions are present between the metal centers in this temperature range.

**Figure 3:**
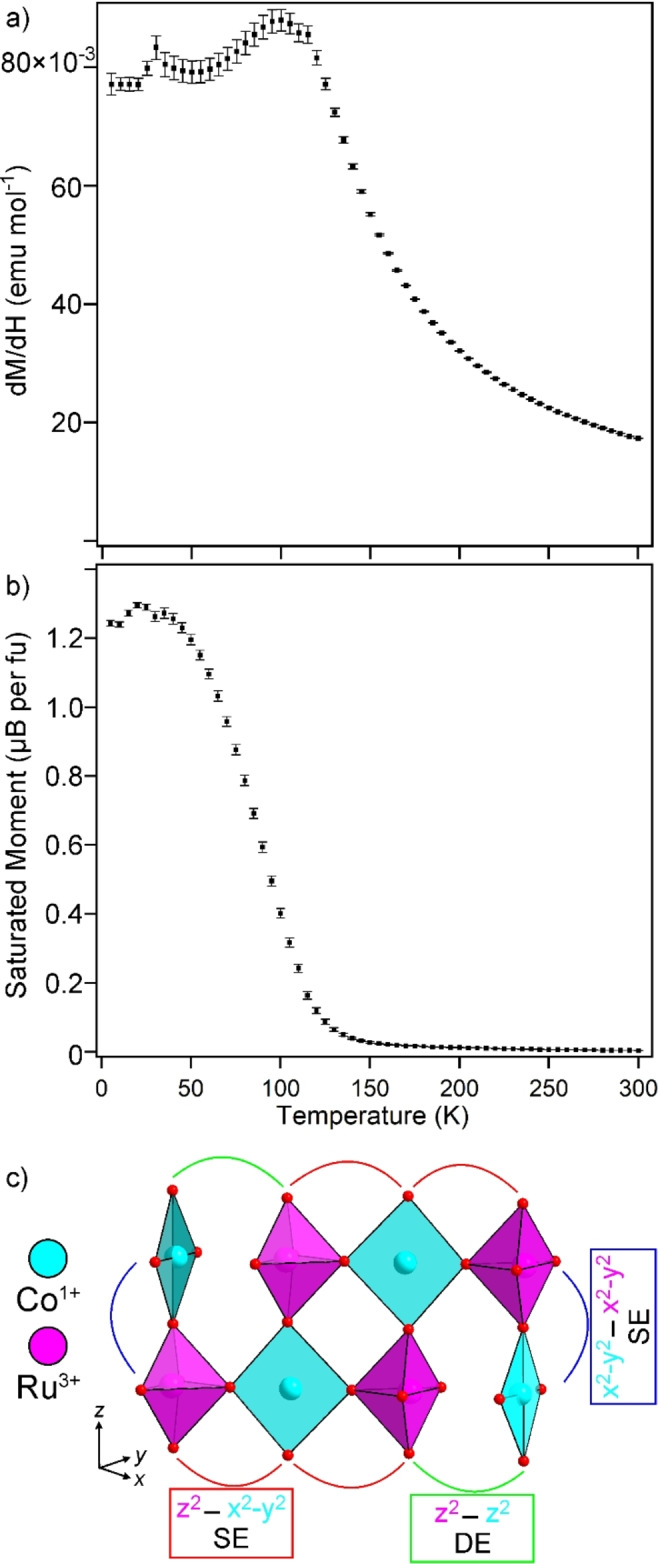
a) Paramagnetic susceptibility and b) saturated ferromagnetic moment of LaSrCoRuO_5_ measured via the ‘ferrosubtraction’ method and plotted as a function of temperature, c) The direct exchange and super exchange pathways in LaSrCoRuO_5_.

On cooling below 120 K there is a large increase in the saturated ferromagnetic moment of the samples (Figure 3b), increasing from 0.03 μB per fu at 150 K (a value that is attributed to the presence of an elemental Co impurity) to ≈1.25 μB per fu at 2 K, indicative of a ferromagnetic state, however NPD data collected at 5 K show no additional features indicative of magnetic order, as described in the Supporting Information.

The bond lengths of the square‐planar and octahedral cobalt sites in LaSrCoRuO_5_ are consistent with a high spin, S=1 Co^1+^ center, and a low spin, S=0 Co^3+^ respectively. Thus, the most significant magnetic couplings in the system will be between the square‐planar Co^1+^ centers, which have a (d_xz/yz_)^4^(d_xy_)^2^(d_z2_)^1^(d_x2−y2_)^1^ electronic configuration, and the Ru^3+^ centers located in square‐based pyramidal sites which have a (d_xz/yz_)^4^(d_xy_)^1^(d_z2_)^0^(d_x2−y2_)^0^ electronic configuration.

As shown in Figure 3c, the Co^1+^ and Ru^3+^ centers are magnetically coupled by either (Ru4d_x2−y2_)−O2p−(Co3d_x2−y2_) or (Ru4d_z2_)−O2p‐(Co3d_x2−y2_) σ‐type super exchange or (Ru4d_z2_)−(Co3d_z2_) direct exchange. Given that the Ru 4d_x2−y2_ and 4d_z2_ orbitals are empty and the corresponding Co3d orbitals are half filled, all of these interactions will be ferromagnetic,^[23]^ consistent with the low‐temperature magnetization data.

The disproportionation of Co^2+^ evident in LaSrCoRuO_5_ is surprising. As noted above, other transition metal cations with d^7^ electron counts (e.g., Pd^3+^, Pt^3+^, Rh^2+^) are observed to disproportionate in oxide environments, driven by the presence of ‘preferred’ coordination sites. However, to date, this behavior has been restricted to 4d and 5d transition metals where the stronger ligand fields (compared to 3d metals) provide a larger energetic stabilization for the d^6^ octahedral and d^8^ square‐planar electron‐count/coordination combinations. It is therefore unexpected to see Co^2+^, a common oxidation state with a modest ligand field in oxides, undergo a coordination‐site driven disproportionation.

There are limited examples of 3d transition metal cations, such as Fe^4+^ and Ni^3+^ disproportionating in extended oxides. However, in these cases the disproportionation of the metal center (e.g. Fe^4+^ in CaFeO_3_ or BaFeO_3_; Ni^3+^ in TlNiO_3_)[^24^, ^25^, ^26^] is driven by a metal‐insulator phase transition driven by the presence of a single electron in the σ‐band of these oxides phases, rather than coordination site preference.

The unique observation of coordination‐site driven disproportionation of Co^2+^ in LaSrCoRuO_5_ suggests that the topochemical reaction which forms LaSrCoRuO_5_ may act to ‘select’ this phase, as the disproportionated structure is a local energy minimum in composition‐structure space in the reaction path between LaSrCoRuO_6_ and LaSrCoRuO_4_. Indeed, the same argument can be applied to the topochemical reactions which form the Rh^I^/Rh^III^ disproportionated phases reported previously.[^6^, ^7^] In combination these observations suggest further coordination‐site driven disproportionated oxide phases could be accessible by this type of low‐temperature reaction, presenting an opportunity to prepare a range of transition metal oxides with a rich variety of novel metal oxidation‐state/coordination geometry‐combinations.

## Conflict of interest

The authors declare no conflict of interest.

## Supporting information

As a service to our authors and readers, this journal provides supporting information supplied by the authors. Such materials are peer reviewed and may be re‐organized for online delivery, but are not copy‐edited or typeset. Technical support issues arising from supporting information (other than missing files) should be addressed to the authors.

Supporting Information

## Data Availability

The data that support the findings of this study are available from the corresponding author upon reasonable request.
