# Supplementary material for: Disproportionation of Co2+ in the Topochemically Reduced Oxide LaSrCoRuO5
Source: Angew Chem Int Ed Engl. 2024 Jan 4;63(6):e202313067. doi: 10.1002/anie.202313067 (PMC10952446; doi:10.1002/anie.202313067)
Supplement: Supplementary file 1 — Supporting Information [file ANIE-63-0-s001.pdf]

Angewandte Chemie
Eine Zeitschrift der Gesellschaft Deutscher Chemiker

Supporting Information

Disproportionation of Co^{2+} in the Topochemically Reduced Oxide $LaSrCoRuO_5$

*Z. Liang, M. Batuk, F. Orlandi, P. Manuel, J. Hadermann, M. A. Hayward**

Supporting Information

Table of Contents

1. Sample Preparation

Synthesis of $LaSrCoRuO_6$.

Figure S1. Observed, calculated and difference plots from the structural refinement of a $P2_1/n$ symmetry model against SXRD data collected from $LaSrCoRuO_6$ at room temperature.

Table S1. Parameters extracted from the structural refinement of slow-cooled and quenched samples of $LaSrNiRuO_6$ against SXRD data.

Reduction of $LaSrCoRuO_6$.

2. Characterization

3. Thermogravimetric Analysis of $LaSrCoRuO_{6-x}$

Figure S2. Thermogravimetric data collected while heating $LaSrCoRuO_{6-x}$ under flowing oxygen.

4. Structural Characterization of $LaSrCoRuO_5$

Figure S3. Observed calculated and difference plots from the structural refinement of $LaSrCoRuO_5$ against SXRD data using a small unit cell (*a* = 5.4064 Å, *b* = 5.4115 Å *c* = 8.1626(1) Å, γ = 90.55(1) °)

Figure S4. Observed calculated and difference plots from the structural refinement of $LaSrCoRuO_5$ against SXRD data using a large unit cell (*a* = 10.8128(2) Å, *b* = 10.8231(2) Å, *c* = 8.1626(1) Å, γ = 90.55(1) °).

Figure S5. Electron diffraction data showing $2\sqrt{2} \times 2\sqrt{2} \times 2$ cell expansion compared to simple ABO_3 perovskite.

Figure S6. Observed, calculated and difference plots from the structural refinement of $LaSrCoRuO_5$ against NPD data collected at room temperature.

Table S2. Parameters from the structural refinement of $LaSrCoRuO_5$ against NPD data collected at 298 K.

Table S3. Selected bond lengths from the refined structure of $LaSrCoRuO_5$ refined against NPD data collected at 298K.

5. Magnetic measurements in the presence of elemental Co impurities via the 'ferrosubtraction' method.

Figure S7. Magnetization of $LaSrCoRuO_5$ measured as a function of applied field at 300 K. A linear fit to high-field region (H > 25000 Oe) yields a gradient which is the paramagnetic susceptibility of the sample, and an intercept which is the saturated ferromagnetic moment of the sample.

6. Magnetic Characterization of $LaSrCoRuO_5$

Figure S8. Magnetization-field isotherm collected from $LasrCoRuO_5$ at 300 K (top), expanded region around zero applied field to highlight hysteresis.

Figure S9. Magnetization-field isotherm collected from $LasrCoRuO_5$ at 5 K .

Figure S10. Zero-field cooled (ZFC) and field cooled (FC) magnetization data collected from $LaSrCoRuO_5$ in an applied field of 100 Oe. Lower panel shows expanded view of high temperature region to highlight divergence between ZFC and FC data due to presence of ferromagnetic impurity.

Figure S11. Plot of 1/ZFC data (from data plotted Figure S10) against temperature showing high temperature region is non-linear due to presence of ferromagnetic impurity.

7. Low-temperature structural characterization.

Figure S12. A comparison of NPD data collected from $LaSrCoRuO_5$ at room temperature and 5 K.
Figure S13. Observed, calculated and difference plots from the structural refinement of $LaSrCoRuO_5$ against NPD data collected at 5 K.
Table S4. Parameters from the structural refinement of $LaSrCoRuO_5$ against NPD data collected at 5 K.

8. EELs data

Figure S14. EELS survey spectrum (left) and Co edges (right) collected from $LaSrCoRuO_5$.
Table S5. Cobalt edge positions and intensity ratios obtained from EELS data collected from $LaSrCoRuO_5$.

1. Sample Preparation

Synthesis of $LaSrCoRuO_6$

Samples of $LaSrCoRuO_6$ were prepared via a citrate gel method. Suitable stoichiometric ratios of La_2O_3 (99.999%), $SrCO_3$ (99.99%), Co powder (99.996%), and RuO_2 (99.99% dried at 800°C) were dissolved in a minimum quantity of 6 M nitric acid. 3 mole equivalents of citric acid and 5 ml of analar ethylene glycol were added and the solution heated with constant stirring. The gels thus formed were subsequently ground into a fine powder, placed in an alumina crucible and heated at 1 °C min^{-1} to 900 °C in air. The powders were then reground, pressed into 13 mm pellets and then heated at 1100 °C in air for 2 periods of 24 h with intermediate regrinding. At the end of the final heating period samples were cooled at a rate of 5 °C min^{-1} to 450 °C and then removed from the furnace and rapidly transferred to a dry-ice-cooled alumina crucible and allowed to rapidly cool.

Synchrotron X-ray powder diffraction data collected from $LaSrCoRuO_6$ could be fit by a structural model previously reported for the phases (space group $P2_1/n$) to achieve a good fit as shown in Figure S1, to yield a crystal structure described in Table S1 in good agreement with previous reports [11, 12].

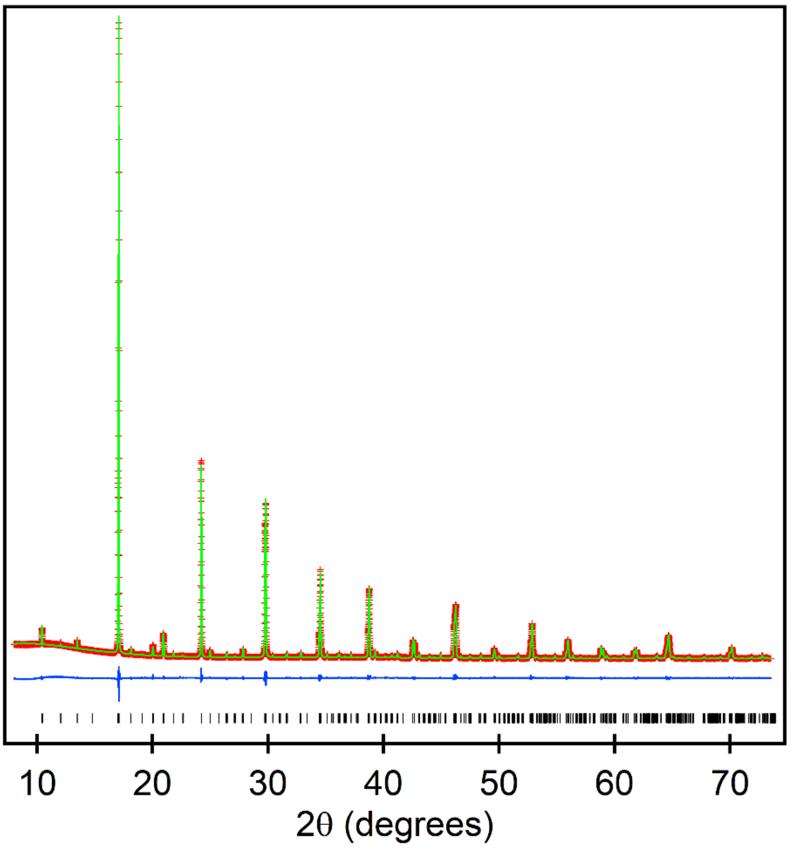

Figure S1. Observed, calculated and difference plots from the structural refinement of a $P2_1/n$ symmetry model against SXRD data collected from $LaSrCoRuO_6$ at room temperature.

LaSrCoRuO$_6$					
Atom	*x*	*y*	*z*	Fraction	B$_{iso}$
La/Sr	0.0055(1)	0.0229(1)	0.2507(1)	0.5/0.5	0.074(5)
Ni/Ru(1)	½	0	½	0.981(3)/0.019(3)	0.033(2)
Ru/Ni(2)	½	0	0	0.981(3)/0.019(3)	0.033(2)
O(1)	0.287(1)	0.278(1)	0.032(1)	1	0.076(4)
O(2)	0.232(1)	0.775(1)	0.027(1)	1	0.076(4)
O(3)	0.933(1)	0.494(1)	0.255(1)	1	0.076(4)
Space Group $P2_1/n$<br>Lattice Parameters<br>a = 5.5909(1) Å, b = 5.5665(1) Å, c = 7.8787(1) Å, β = 89.993(1),<br>volume = 245.20(1) Å^3					
Radiation source: Synchrotron X-ray, λ = 0.8268 Å<br>Temperature: 298 K<br>Rp = 3.30%, Rwp = 4.27%, R$_{Bragg}$ = 1.33%					

Table S1. Parameters extracted from the structural refinement of LaSrCoRuO$_6$ against SXRD data.

Reduction of LaSrCoRuO$_6$

Samples of LaSrCoRuO$_6$ were topochemically reduced to LaSrCoRuO$_5$ using Zr as a reducing agent. A series of test reactions were performed in which 200 mg samples of LaSrCoRuO$_6$ was sealed in an evacuated silica ampoules along with a glass 'thimble' containing 2 mole equivalents of powdered zirconium, such that the two powders shared an atmosphere but were not in physical contact. The apparatus was then heated for 36 h at temperatures between 400 < T/ °C < 530 to assess reactivity. The optimum reaction temperature was observed to be 450 °C. X-ray diffraction data collected from products of reactions performed below this temperature were observed to exhibit broad diffraction peaks, while data from the products of reactions performed at higher temperature contained a secondary phase tentatively assigned as LaSrCoRuO$_4$.

A sample of LaSrCoRuO$_5$ suitable for neutron diffraction analysis was prepared by sealing 1.5 g of LaSrCoRuO$_6$ in a silica ampoule along with a glass 'thimble' containing 2 mole equivalents of powdered zirconium, such that the two powders shared an atmosphere but were not in physical contact. This apparatus was heated at 450 °C for one period of 48 hours. The apparatus was then allowed to cool to room temperature and was then opened in an argon-filled glove box, and reground before further analysis.

2. Characterization

Reaction progress and initial structural characterization was performed using laboratory X-ray powder diffraction (PXRD) data collected using a PANalytical X'pert diffractometer incorporating an X'celerator position-sensitive detector (monochromatic Cu Kα1 radiation). Data were collected from reduced samples using home-made air tight holders in which samples had been loaded from within an argon-filled glove box.

High-resolution synchrotron X-ray powder diffraction (SXRD) data were collected using the I11 instrument at the Diamond Light Source Ltd. Diffraction patterns were collected using Si-calibrated X-rays with an approximate wavelength of 0.825 Å from samples, sealed in 0.3 mm diameter borosilicate glass capillaries.

Neutron powder diffraction (NPD) data were collected from samples contained within vanadium cans sealed under an inert atmosphere at room temperature and 5K using the WISH diffractometer at the ISIS neutron source, UK. Rietveld refinement of powder diffraction data was performed using the TOPAS Academic (V6).[16]

Three-dimensional electron diffraction (3D ED) data were acquired at a FEI Titan 80-300 "cubed" microscope operated at 300 kV. The specimens for the TEM study were prepared by grinding the material in ethanol and depositing a few drops of the suspension onto a copper TEM grid covered by a continuous carbon layer. The specimens were prepared in an Ar-filled glove box. Samples were analyzed both using a Fischione tomography single-tilt holder with a tilting range -70 to 70° (in this case the specimens were exposed to air for about 1 minute) and using a Gatan vacuum transfer holder with a tilting range -40 to 40° (no contact with air). No difference was observed, confirming that the materials are stable in air for at least a short time. The materials were stable under the electron beam. ED patterns were obtained in an automated manner with 0.5° tilt intervals using fast-ADT software.[13] The PETS2.0 [14] software was used to analyze the data and make the 3D reconstruction of the reciprocal space.

Thermogravimetric analysis (TGA) measurements were performed by heating powder samples under flowing oxygen, using a Mettler-Toledo MX1 thermogravimetric microbalance.

DC magnetization data were collected using a Quantum Design MPMS SQUID magnetometer from samples contained in gelatin capsules using the 'ferrosubtract' method described in section 5 below.

3. Thermogravimetric Analysis of $LaSrCoRuO_{6-x}$

Thermogravimetric data were collected while heating a sample of $LaSrCoRuO_{6-x}$ under flowing oxygen at a rate of 5 °C min^{-1} to 800 °C are shown in Figure S1. These data show a mass increase of 3.2% as $LaSrCoRuO_{6-x}$ was reoxidized back to $LaSrCoRuO_6$ (confirmed by X-ray powder diffraction) indicating an initial composition of $LaSrCoRuO_{5.02(3)}$.

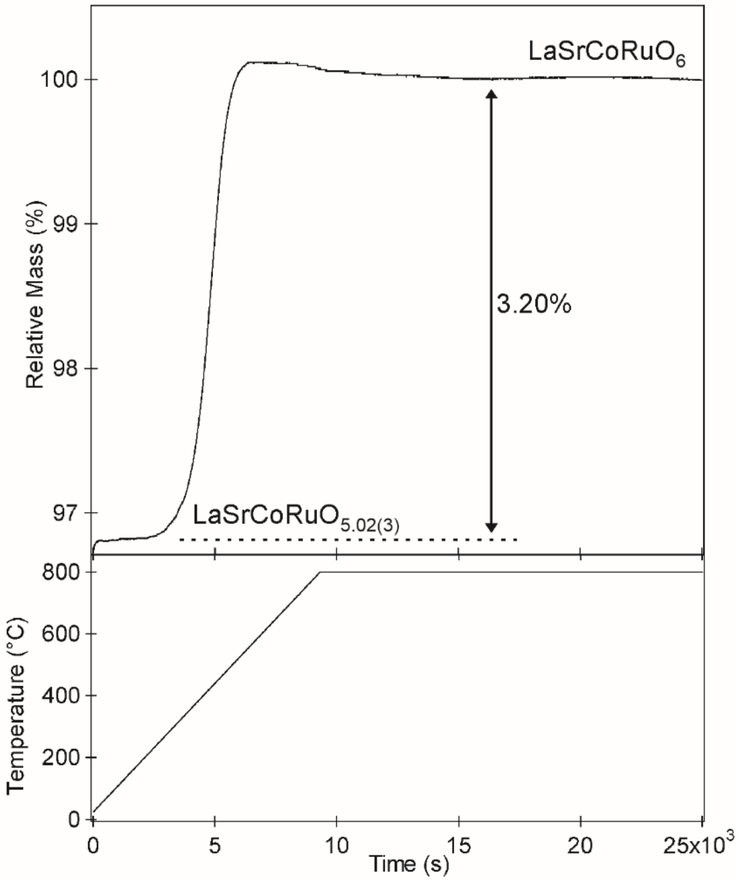

Figure S2. Thermogravimetric data collected while heating $LaSrCoRuO_{6-x}$ under flowing oxygen.

4. Structural Characterization of $LaSrCoRuO_5$

SXRD data collected from $LaSrCoRuO_5$ could be indexed using a body-centered monoclinic unit cell (a = 5.40 Å, b = 5.41 Å, c = 8.16 Å, γ = 90.5 °) consistent with the retention of the perovskite framework from the $LaSrCoRuO_6$ parent phase. However, close inspection revealed a series of weak additional reflections in the SXRD data that could not be indexed by this cell as shown in Figure S3.

Electron diffraction data collected from $LaSrCoRuO_5$ (Figure S5) is consistent with a 2 × 2 × 1 cell expansion compared to the $LaSrCoRuO_6$ parent phase (2√2 × 2√2 × 2 compared to a simple ABO_3 perovskite unit cell). This expanded cell accounts for all the additional weak peaks observed in the SXRD data (Figure S4) and can also index NPD data collected at room temperature from $LaSrCoRuO_5$ (Figure S6)

Considering the $A_2BB'O_5$ composition and the 2√2 × 2√2 × 2 cell expansion of the phase, a number of anion-vacancy ordered and B-site cation ordered perovskite structural models were considered for $LaSrCoRuO_5$. It was observed that a good fit to the SXRD and NPD data could be achieved using a model based on the anion-vacancy ordered structure of $LaNi_{0.9}Co_{0.1}O_{2.5}$ which consists of a network of apex-linked 6-coordinate octahedral, 5-coordinate square-based pyramidal and 4-coordinate square planar BO_x units.[15] The model was modified to take account of the rock salt ordering of the Co and Ru cations, so that the Ru centers were exclusively located within 5-coordinate sites, while the Co centers occupied both 6- and 4-coordinate sites within a monoclinic unit cell (a = 10.8128(2) Å, b = 10.8231(2) Å, c = 8.1626(1) Å, γ = 90.55(1) °) with $P112_1$ space group symmetry. The model was refined against the NPD data to achieve a good fit (wRp = 6.33%) as shown in Figure S6 and described in detail in Table S2, with selected bond lengths given in Table S3.

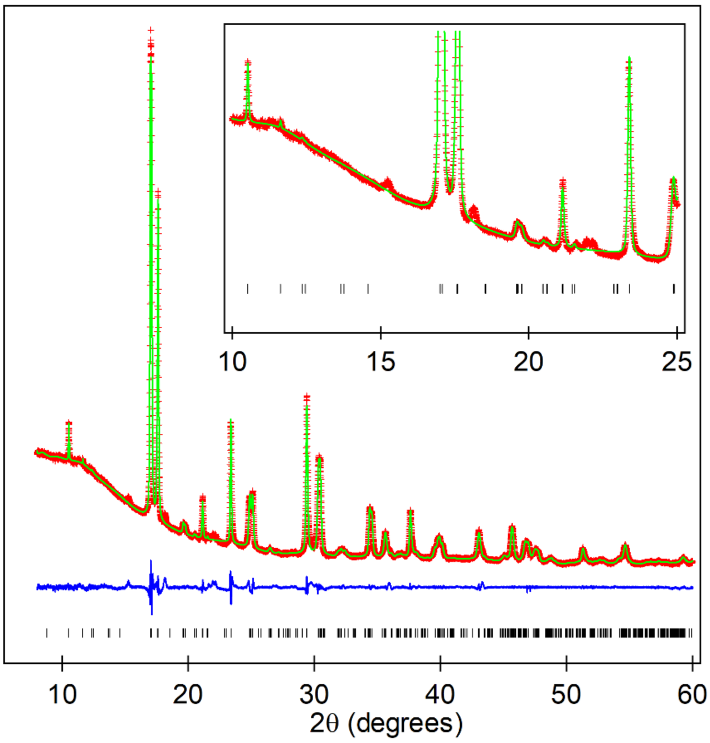

Figure S3. Observed calculated and difference plots from the structural refinement of $LaSrCoRuO_5$ against SXRD data using a small unit cell (*a* = 5.4064 Å, *b* = 5.4115 Å *c* = 8.1626(1) Å, γ = 90.55(1) °)

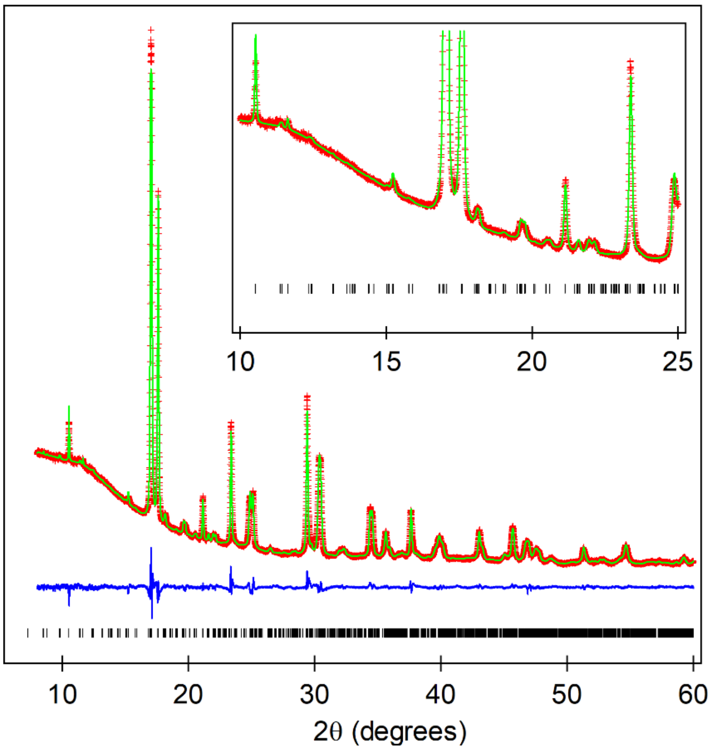

Figure S4. Observed calculated and difference plots from the structural refinement of $LaSrCoRuO_5$ against SXRD data using a large unit cell (*a* = 10.8128(2) Å, *b* = 10.8231(2) Å, *c* = 8.1626(1) Å, γ = 90.55(1) °).

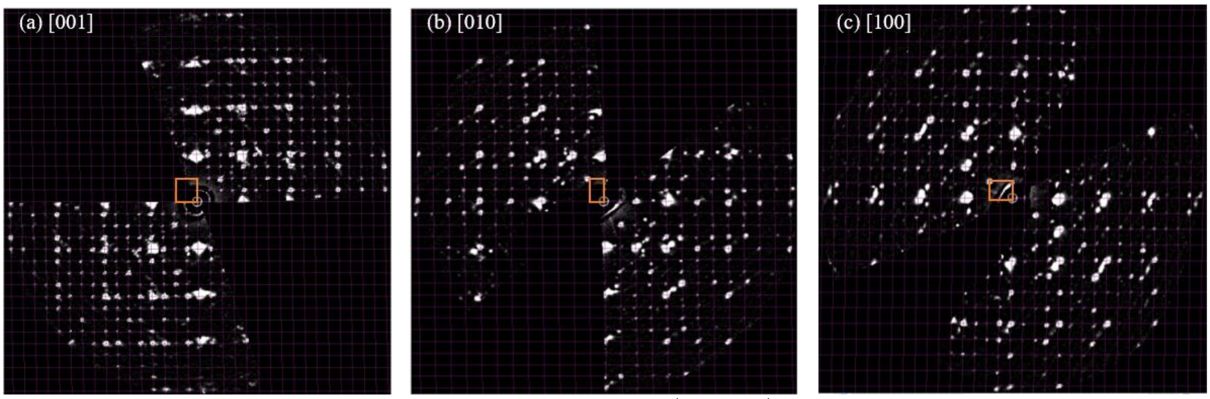

Figure S5. Electron diffraction data showing 2√2 × 2√2 × 2 cell expansion compared to simple ABO_3 perovskite.

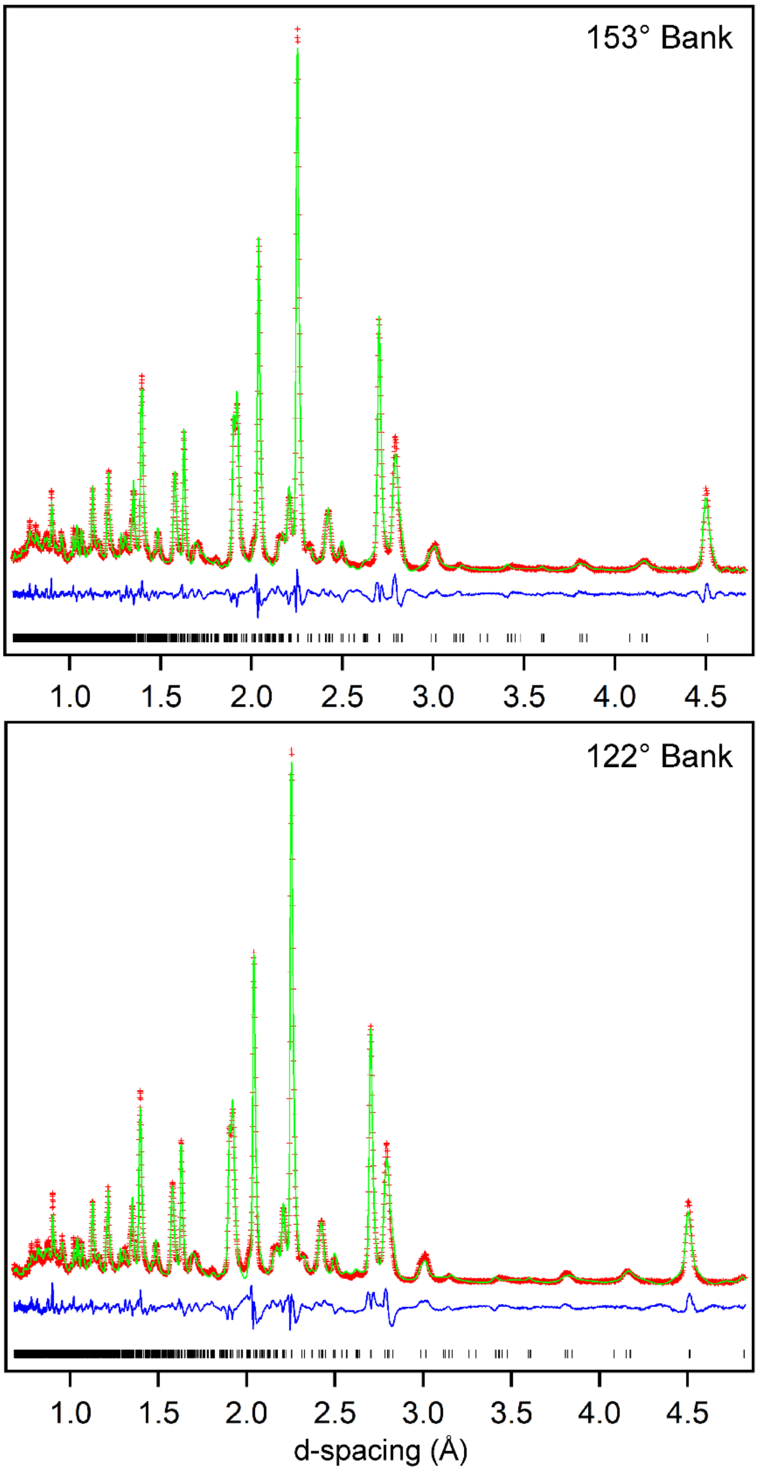

Figure S6. Observed, calculated and difference plots from the structural refinement of $LaSrCoRuO_5$ against NPD data collected at room temperature.

Atom	*x*	*y*	*z*	Fraction	B_{iso}
Co(1)	$^5/_8$	$^5/_8$	$^3/_4$	1	0.82(2)
Co(2)	0.612(3)	0.136(3)	$^3/_4$	1	0.82(2)
Co(3)	$^1/_8$	$^1/_8$	$^3/_4$	1	0.82(2)
Co(4)	0.136(3)	0.612(3)	$^3/_4$	1	0.82(2)
Ru(1)	0.3648(7)	0.3648(7)	$^3/_4$	1	2.26(12)
Ru(2)	0.8707(8)	0.3793(8)	$^3/_4$	1	2.26(12)
Ru(3)	0.8852(7)	0.8852(7)	$^3/_4$	1	2.26(12)
Ru(4)	0.3793(8)	0.8707(8)	$^3/_4$	1	2.26(12)
O(1)	0.0334(8)	0.7865(8)	$^3/_4$	1	3.14(7)
O(2)	0.9679(10)	0.5359(9)	$^3/_4$	1	3.14(7)
O(3)	0.2821(10)	0.7141(9)	$^3/_4$	1	3.14(7)
O(4)	0.2166(8)	0.4635(8)	$^3/_4$	1	3.14(7)
O(5)	0.7141(9)	0.2821(10)	$^3/_4$	1	3.14(7)
O(6)	0.7865(8)	0.0334(8)	$^3/_4$	1	3.14(7)
O(7)	0.4635(8)	0.2166(8)	$^3/_4$	1	3.14(7)
O(8)	0.5359(9)	0.9679(9)	$^3/_4$	1	3.14(7)
O(9)	0.4922(10)	0.4922(10)	$^3/_4$	1	3.14(7)
O(10)	0.7578(10)	0.7578(10)	$^3/_4$	1	3.14(7)
O(11)	0.9923(10)	0.2577(10)	$^3/_4$	1	3.14(7)
O(12)	0.2577(10)	0.9923(10)	$^3/_4$	1	3.14(7)
O(13)	0.8819(6)	0.8819(6)	0.5093(11)	1	3.14(7)
O(14)	0.8819(6)	0.8819(6)	0.9907(11)	1	3.14(7)
O(15)	0.3681(6)	0.3681(6)	0.9907(11)	1	3.14(7)
O(16)	0.3681(6)	0.3681(6)	0.5093(11)	1	3.14(7)
O(17)	0.6069(16)	0.1431(16)	0.0001(10)	1	3.14(7)
O(18)	0.6193(19)	0.1307(19)	0.4999(10)	1	3.14(7)
O(19)	0.1431(16)	0.6069(16)	0.0001(10)	1	3.14(7)
O(20)	0.1307(19)	0.6193(19)	0.4999(10)	1	3.14(7)
La/Sr(1)	0.108(3)	0.371(4)	0	0.5/0.5	1.61(6)
La/Sr(2)	0.885(4)	0.641(3)	0	0.5/0.5	1.61(6)
La/Sr(3)	0.371(4)	0.108(3)	0	0.5/0.5	1.61(6)
La/Sr(4)	0.641(3)	0.885(4)	0	0.5/0.5	1.61(6)
La/Sr(5)	0.858(4)	0.138(3)	0	0.5/0.5	1.61(6)
La/Sr(6)	0.138(3)	0.858(3)	0	0.5/0.5	1.61(6)
La/Sr(7)	0.382(3)	0.615(3)	0	0.5/0.5	1.61(6)
La/Sr(8)	0.615(3)	0.382(3)	0	0.5/0.5	1.61(6)
$LaSrCoRuO_5$ – Space group $P112_1$ (#4)<br>*a* = 10.8128(2) Å, *b* = 10.8231(2) Å, *c* = 8.1626(1) Å, γ = 90.55(1) °,<br>volume = 955.211(1) $Å^3$					
Radiation source: Neutron Time-of-flight<br>Temperature: 298 K<br>R_p = 5.51 %, wR_p = 6.33 %					

Table S2. Parameters from the structural refinement of $LaSrCoRuO_5$ against NPD data collected at 298 K.

Cation	Anion	Bond length (Å)	BVS	Cation	Anion	Bond length (Å)	BVS
Co(1)	O(9)	2.032(11)	Co+1.42	Co(3)	O(11)	2.030(11)	Co+1.43
	O(10)	2.032(11)			O(12)	2.030(11)	
	O(15)	2.119(9)			O(13)	2.119(9)	
	O(16)	2.119(9)			O(14)	2.119(9)	
Co(2)	O(5)	1.929(11)	Co+2.69	Co(4)	O(1)	2.190(10)	Co+2.69
	O(6)	2.189(10)			O(2)	1.996(12)	
	O(7)	1.827(10)			O(3)	1.928(11)	
	O(8)	1.997(12)			O(4)	1.828(10)	
	O(17)	2.042(1)			O(19)	2.042(1)	
	O(18)	2.043(1)			O(20)	2.043(1)	
Ru(1)	O(4)	1.926(12)	Ru+3.69	Ru(3)	O(1)	1.926(12)	Ru+3.69
	O(7)	1.927(12)			O(6)	1.927(12)	
	O(9)	1.949(14)			O(10)	1.949(14)	
	O(15)	1.965(9)			O(13)	1.965(9)	
	O(16)	1.965(9)			O(14)	1.965(9)	
Ru(2)	O(2)	1.994(14)	Ru+3.35	Ru(4)	O(3)	1.994(14)	Ru+3.35
	O(5)	1.993(14)			O(8)	1.993(14)	
	O(11)	1.860(14)			O(12)	1.860(14)	
	O(19)	2.052(2)			O(17)	2.052(2)	
	O(20)	2.040(1)			O(18)	2.040(1)	

Table S3. Selected bond lengths from the refined structure of $LaSrCoRuO_5$ refined against NPD data collected at 298K.

5. Magnetic measurements in the presence of elemental Co impurities via the 'ferrosubtraction' method

Procedure used to measure the magnetization of samples containing elemental cobalt: The magnetization of elemental Co is observed to saturate in applied magnetic fields of more than 2 T. Thus the paramagnetic susceptibility of a bulk sample can be measured in the presence of elemental Co impurities by measuring the gradient of magnetization-field isotherms in applied fields larger than 2 T. As shown in Figure S7.

To this end the magnetization of samples was measured in a series of 5 fields between 3 T and 5 T. The magnetization vs. field data were fitted to a linear function, the gradient of which is the paramagnetic susceptibility of the bulk sample and the intercept is the saturated ferromagnetic moment of the sample. Data points with large errors were excluded from fits. All fits had at least 4 data points. This procedure was repeated at 5 K intervals between 5 K and 300 K to measure the temperature dependent susceptibility of samples.

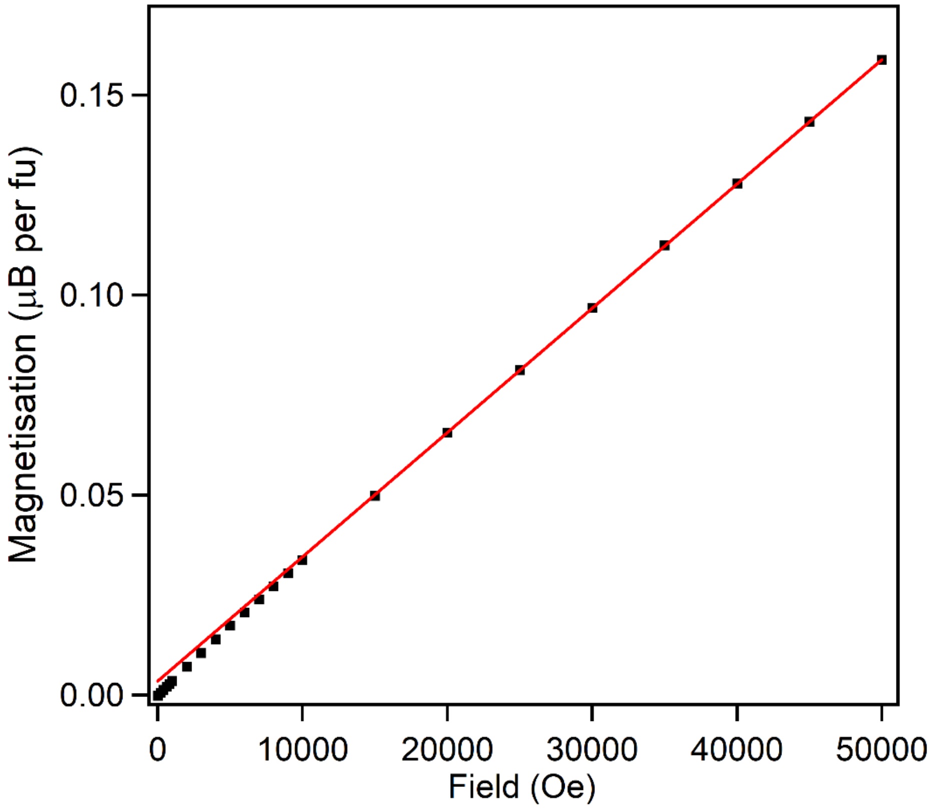

Figure S7. Magnetization of $LaSrCoRuO_5$ measured as a function of applied field at 300 K. A linear fit to high-field region (H > 25000 Oe) yields a gradient which is the paramagnetic susceptibility of the sample, and an intercept which is the saturated ferromagnetic moment of the sample.

6. Magnetic Characterization of $LaSrCoRuO_5$

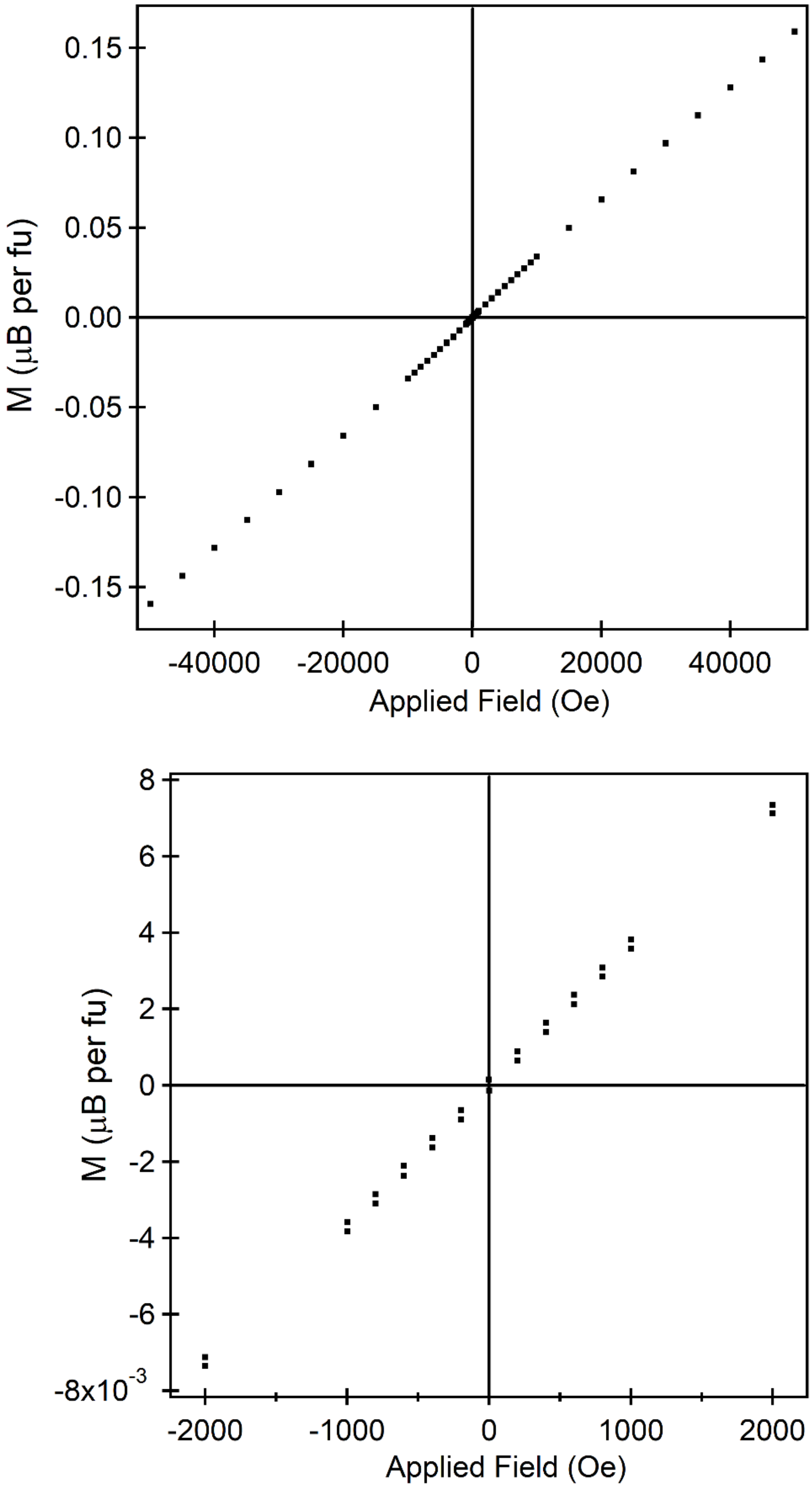

Figure S8. Magnetization-field isotherm collected from $LasrCoRuO_5$ at 300 K (top), expanded region around zero applied field to highlight hysteresis (bottom).

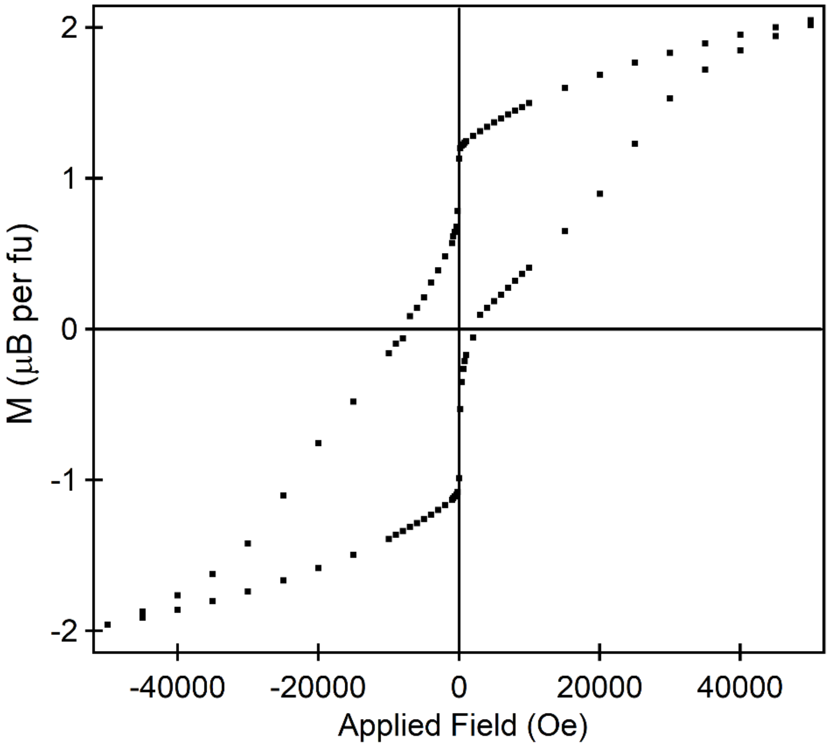

Figure S9. Magnetization-field isotherm collected from LasrCoRuO_5 at 5 K .

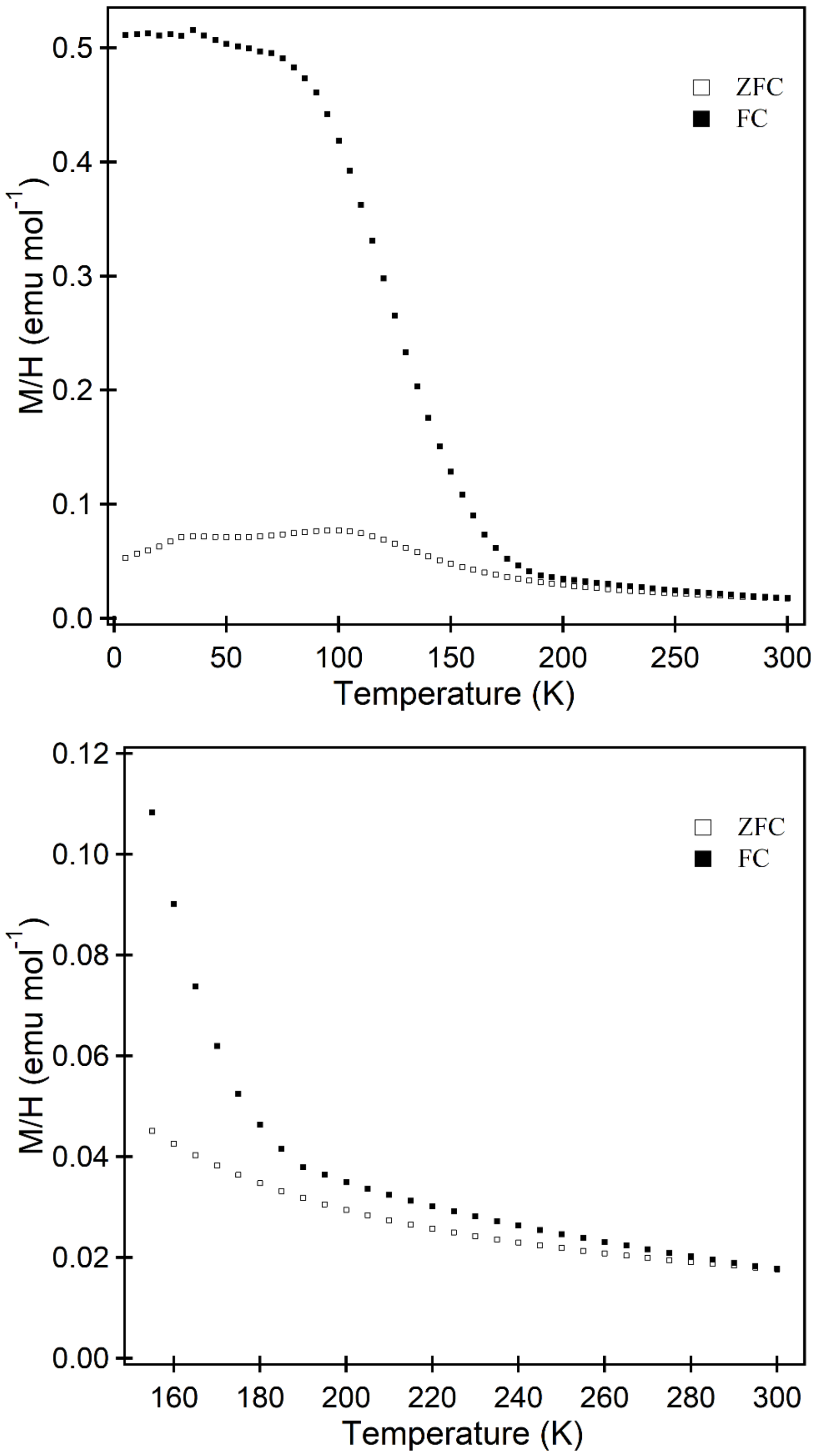

Figure S10. Zero-field cooled (ZFC) and field cooled (FC) magnetization data collected from $LaSrCoRuO_5$ in an applied field of 100 Oe. Lower panel shows expanded view of high temperature region to highlight divergence between ZFC and FC data due to presence of ferromagnetic impurity.

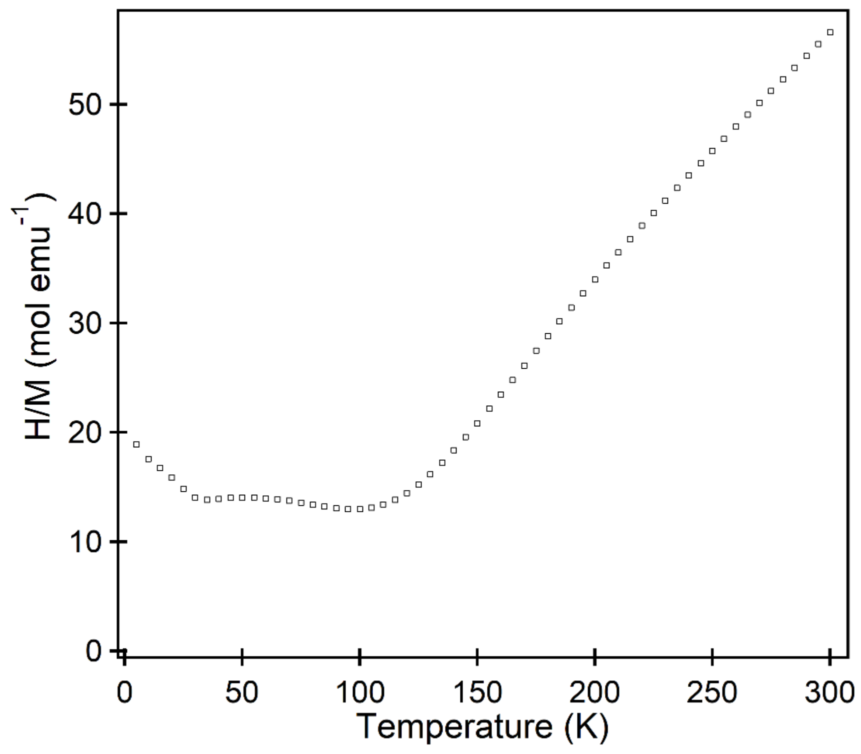

Figure S11. Plot of 1/ZFC data (from data plotted Figure S10) against temperature showing high temperature region is non-linear due to presence of ferromagnetic impurity.

7. Low-temperature structural characterization.

Comparison of NPD data collected from $LaSrCoRuO_5$ at room temperature and 5 K (Figure S12) show no obvious differences.

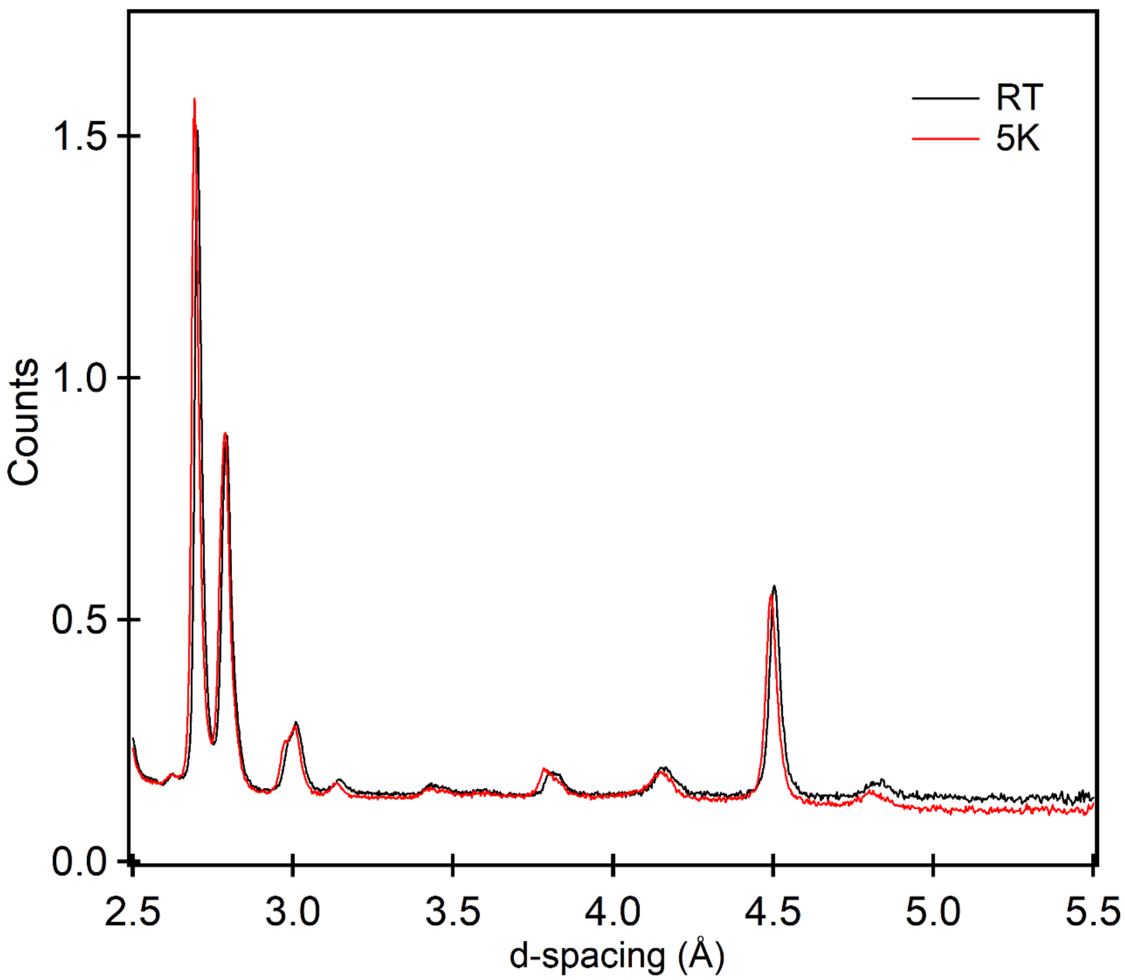

Figure S12. A comparison of NPD data collected from $LaSrCoRuO_5$ at room temperature and 5 K.

The lack of magnetic scattering features in the NPD data collected from $LaSrCoRuO_5$ in zero-applied magnetic field suggests the ferromagnetic behavior observed in the magnetization data could only be short-range ordered in the absence of an applied magnetic field. A nuclear-only structural model was refined against these data to give a good fit, as shown below.

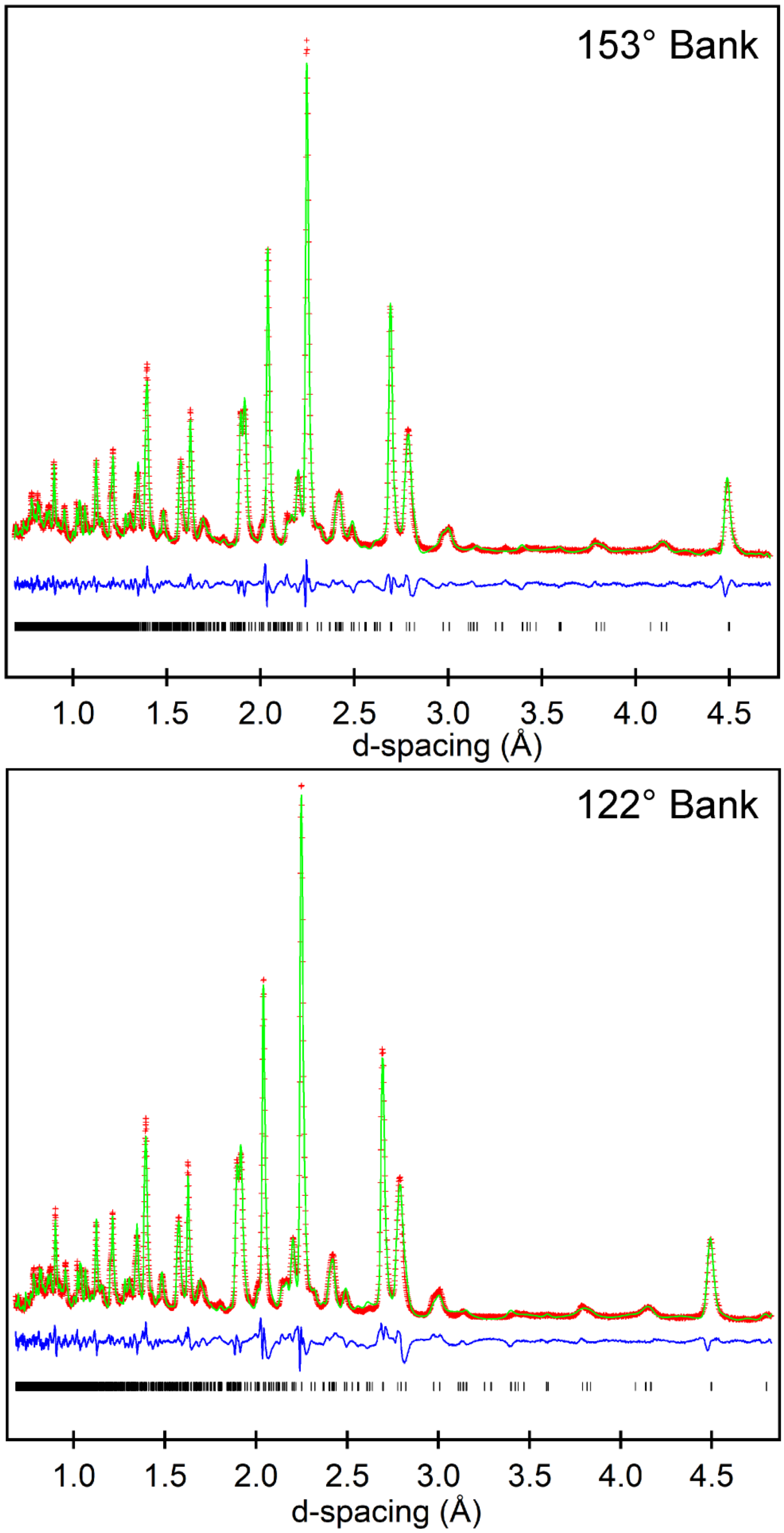

Figure S13. Observed, calculated and difference plots from the structural refinement of $LaSrCoRuO_5$ against NPD data collected at 5 K.

Atom	*x*	*y*	*z*	Fraction	B_{iso}
Co(1)	$^5/_8$	$^5/_8$	$^3/_4$	1	0.64(2)
Co(2)	0.615(3)	0.136(3)	$^3/_4$	1	0.64(2)
Co(3)	$^1/_8$	$^1/_8$	$^3/_4$	1	0.64(2)
Co(4)	0.136(3)	0.615(3)	$^3/_4$	1	0.64(2)
Ru(1)	0.3643(5)	0.3643(5)	$^3/_4$	1	0.67(9)
Ru(2)	0.8709(6)	0.3791(6)	$^3/_4$	1	0.67(9)
Ru(3)	0.8857(5)	0.8857(5)	$^3/_4$	1	0.67(9)
Ru(4)	0.3791(6)	0.8709(6)	$^3/_4$	1	0.67(9)
O(1)	0.0334(8)	0.7864(8)	$^3/_4$	1	1.80(5)
O(2)	0.9670(10)	0.5359(9)	$^3/_4$	1	1.80(5)
O(3)	0.2821(10)	0.7141(9)	$^3/_4$	1	1.80(5)
O(4)	0.2166(8)	0.4635(8)	$^3/_4$	1	1.80(5)
O(5)	0.7141(9)	0.2821(10)	$^3/_4$	1	1.80(5)
O(6)	0.7864(8)	0.0334(8)	$^3/_4$	1	1.80(5)
O(7)	0.4635(8)	0.2166(8)	$^3/_4$	1	1.80(5)
O(8)	0.5359(9)	0.9670(10)	$^3/_4$	1	1.80(5)
O(9)	0.4920(8)	0.4920(8)	$^3/_4$	1	1.80(5)
O(10)	0.7580(8)	0.7580(8)	$^3/_4$	1	1.80(5)
O(11)	0.9940(8)	0.2560(8)	$^3/_4$	1	1.80(5)
O(12)	0.2560(8)	0.9940(8)	$^3/_4$	1	1.80(5)
O(13)	0.8819(6)	0.8819(6)	0.5093(11)	1	1.80(5)
O(14)	0.8819(6)	0.8819(6)	0.9907(11)	1	1.80(5)
O(15)	0.3681(6)	0.3681(6)	0.9907(11)	1	1.80(5)
O(16)	0.3681(6)	0.3681(6)	0.5093(11)	1	1.80(5)
O(17)	0.6070(16)	0.1431(16)	0.9997(10)	1	1.80(5)
O(18)	0.6193(19)	0.1307(19)	0.5003(10)	1	1.80(5)
O(19)	0.1431(16)	0.6070(16)	0.9997(10)	1	1.80(5)
O(20)	0.1307(19)	0.6193(19)	0.5003(10)	1	1.80(5)
La/Sr(1)	0.107(3)	0.371(3)	0	0.5/0.5	0.32(4)
La/Sr(2)	0.885(3)	0.640(3)	0	0.5/0.5	0.32(4)
La/Sr(3)	0.371(3)	0.107(3)	0	0.5/0.5	0.32(4)
La/Sr(4)	0.640(2)	0.885(3)	0	0.5/0.5	0.32(4)
La/Sr(5)	0.858(3)	0.138(3)	0	0.5/0.5	0.32(4)
La/Sr(6)	0.138(3)	0.858(3)	0	0.5/0.5	0.32(4)
La/Sr(7)	0.382(3)	0.615(3)	0	0.5/0.5	0.32(4)
La/Sr(8)	0.615(3)	0.382(3)	0	0.5/0.5	0.32(4)
$LaSrCoRuO_5$ – Space group $P112_1$ (#4)<br>a = 10.776(3) Å, b = 10.785(3) Å, c = 8.1598(1) Å, γ = 90.65(1) °,<br>volume = 955.211(1) $Å^3$					
Radiation source: Neutron Time-of-flight<br>Temperature: 5 K<br>R_p = 4.91 %, wR_p = 5.93 %					

Table S4. Parameters from the structural refinement of $LaSrCoRuO_5$ against NPD data collected at 5 K.

8. EELs data

Mono STEP EELS data were collected using an FEI Titan[3] instrument operating at 300 kV with an energy resolution of 0.4 eV and a convergence angle of 18.06 mrad.

The sample was prepared under an argon atmosphere by drop casting the powder in an ethanol suspension onto a Cu grid coated in holey carbon.

DUAL EELS data were taken for all spectra (zero-loss peak and the Co edge) for calibration. In total, EELS spectra of the Co edge were taken from 13 crystals, from which 8 had a sufficiently small thickness to show a distinct Co L_2 peak. The latter ones were included in the quantitative table (see below). The EELS spectra were always averaged over particle (edge) areas between 100 nm^2 and 2000 nm^2 in size, within which no Co peak position variation was observed from the edge further into the bulk of the crystal.

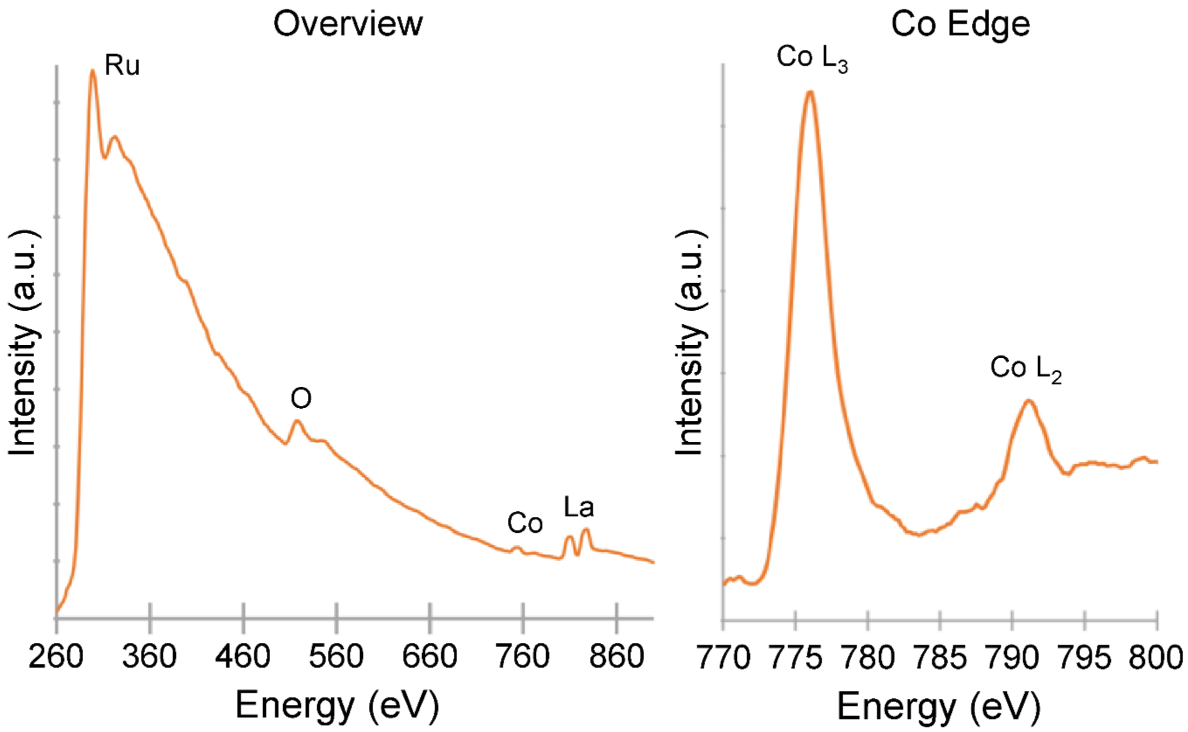

Figure S14. EELS survey spectrum (left) and Co edges (right) collected from $LaSrCoRuO_5$.

**Crystal no.**	**L3 – L2 peak difference (eV)**	**L3 – L2 intensity ratio**
3	14.9(4)	4.85
4	15.1(4)	5.01
5	15.2(4)	5.01
7	15.1(4)	4.48
9	15.1(4)	5.43
10	15.0(4)	5.18
12	15.1(4)	4.43
13	15.0(4)	4.27
Average	15.0625	4.83
Standard dev.	0.086	0.40

Table S5. Cobalt edge positions and intensity ratios obtained from EELS data collected from $LaSrCoRuO_5$.
